# Current and next-year cranberry yields predicted from local features and carryover effects

**DOI:** 10.1371/journal.pone.0250575

**Published:** 2021-05-10

**Authors:** Léon Etienne Parent, Reza Jamaly, Amaya Atucha, Elizabeth Jeanne Parent, Beth Ann Workmaster, Noura Ziadi, Serge-Étienne Parent

**Affiliations:** 1 Département des Sols et de Génie Agroalimentaire, Université Laval, Québec, Québec, Canada; 2 Departamento de Solos, Universidade Federal de Santa Maria, Camobi - Santa Maria, Rio Grande do Sul, Brazil; 3 Department of Horticulture, College of Agriculture and Life Sciences, University of Wisconsin-Madison, Madison, Wisconsin, United States of America; 4 Quebec Research and Development Centre, Agriculture and Agri-Food Canada, Québec, Québec, Canada; Federal University of Mato Grosso do Sul, BRAZIL

## Abstract

Wisconsin and Quebec are the world leading cranberry-producing regions. Cranberries are grown in acidic, naturally low-fertility sandy beds. Cranberry fertilization is guided by general soil and tissue nutrient tests in addition to yield target and vegetative biomass. However, other factors such as cultivar, location, and carbon and nutrient storage impact cranberry nutrition and yield. The objective of this study was to customize nutrient diagnosis and fertilizer recommendation at local scale and for next-year cranberry production after accounting for local factors and carbon and nutrient carryover effects. We collected 1768 observations from on-farm surveys and fertilizer trials in Quebec and Wisconsin to elaborate a machine learning model using minimum datasets. We tested carryover effects in a 5-year Quebec fertilizer experiment established on permanent plots. Micronutrients contributed more than macronutrients to variation in tissue compositions. Random Forest model related accurately current-year berry yield to location, cultivars, climatic indices, fertilization, and tissue and soil tests as features (classification accuracy of 0.83). Comparing compositions of defective and successful tissue compositions in the Euclidean space of tissue compositions, the general across-factor diagnosis differed from the local factor-specific diagnosis. Nutrient standards elaborated in one region could hardly be transposed to another and, within the same region, from one bed to another due to site-specific characteristics. Next-year yield and nutrient adjustment could be predicted accurately from current-year yield and tissue composition and other features, with R^2^ value of 0.73 in regression mode and classification accuracy of 0.85. Compositional and machine learning methods proved to be effective to customize nutrient diagnosis and predict site-specific measures for nutrient management of cranberry stands. This study emphasized the need to acquire large experimental and observational datasets to capture the numerous factor combinations impacting current and next-year cranberry yields at local scale.

## Introduction

Cranberry (*Vaccinium macrocarpon* Ait.) is an ericaceous plant grown commercially in North America since the 19^th^ century [1]. Cranberry beds, 1–2 ha in size, are established in low-lying position and diked to facilitate water management [2]. Cranberry soils are acidic and vary widely from peaty to sandy [3]. Soil particle arrangement may also change with time, impacting soil hydraulic properties [4,5].

Wisconsin (USA) and Quebec (Canada) are the world leaders in cranberry production. Quebec leads the production of organic cranberries. Hummer et al. [6] reported three periods of cranberry selections for commercial production: native selections, early cultivars, and recent hybrids. Twenty-five cultivars were grown in USA and Canada in 2017. Cranberry response to fertilization is known to be cultivar-, yield-, and region-specific [7–9].

Cranberry fertilization is presently guided by general soil and tissue nutrient tests [10–13] as well as yield and the occurrence of excessive production of vegetative biomass. Cranberry grows best at pH 4.0–5.5 [13]. For perennial crops such as cranbrerry, soil tests are often weakly correlated with fruit yield and are thus complementary to tissue tests [14]. Nevetheless, soil tests provide information on soil’s capacity to supply nutrients. Results of tissue tests are sensitive to genetic and environmental factors [15]. The interpretation of tissue tests is made even more complicated by nutrient interactions [16] and crosstalks [17,18], the fertilization regime [19], soil temperature impacting organic matter decomposition [2], pollination, pests, fruit loading, climatic effects, plant vigor, pruning, irrigation, fruit quality [14], and possibly nutrient uptake by roots in the preceding fall if conditions are favorable [20]. Carryover effects occur where carbohydrates and nutrients accumulated in preceding years impact on yield during the current or next seasons of production. Hence, diagnosis conducted in relation with the current production yield and based only on fixed critical concentration ranges can be misleading [21].

Tissue testing conducted annually is well suited to perennial crops for long-term nutrient management [14]. Change in fertilization regimes may take more than one season to be effective because carbohydrate and nutrient reserves accumulated in off-years can be remobilized at high rate in on-years [22,23]. Alternate bearing caused by resource competition could deplete carbohydrate reserves required to sustain high-production over years [8]. Cranberry fruit set and berry yield are partially limited by carbohydrates, leading to biennial yields that may be attenuated by sanding and pruning cranberry stands [24]. Total amounts of nutrients in fruit plants include mineral elements recycled from previous years and taken up during the current season [23,25–28]. Yield predictions and nutrient requirements for the coming years are important information needed by growers to design fertilization programs close to crop needs.

Cranberry was found to be more responsive to nitrogen than to other nutrients [9,29,30]. While nitrogen fertilization may show no effect on fruit set or cranberry yield during the year of application [31], N overfertilization may result in fewer and poor-quality fruits, and excess vegetative growth may increase plant susceptibility to disease, spring frost, and insect feeding [2,23]. High N rates may produce adverse effects in following years as the N stored in excess is remobilized [2]. However, the observed carryover effects have not been supported by models to predict future yields and nutrient requirements in cranberry agroecosystems.

Because several features impact cranberry mineral nutrition, regional guidelines are likely to be less relevant at local scale where factor interactions occur and numerous factor can be combined succcessfully to produce nutritionally healthy plants. Large and diversified data sets are thus required to capture numerous combinations of growth-impacting factors and to document diagnostic models [32,33]. Methods of artificial intelligence and compositional data analysis can unravel complex patterns that are beyond human capabilities [34,35].

We hypothesized that (1) cranberry tissue compositions can be diagnosed accurately at local scale using a minimum set of yield-impacting features, and (2) cranberry yield prediction is impacted by the preceding yield that reflects prior carbohydrate consumption and by nutrient carryover that reflects prior nutrient storage. Our objective was to customize cranberry nutrient diagnosis in Quebec and Wisconsin. Hypothesis no. 1 was tested using a large dataset collected in Quebec and Wisconsin. Hypothesis no. 2 was tested using a 5-year experiment conducted in central Quebec. Regional and local diagnoses were compared.

## Material and methods

### Datasets

The dataset comprised 1768 observations on tissue composition and berry yield collected from Quebec fertilizer trials at plot scale in 2000–2002 and 2014–2018, and from Quebec and Wisconsin cranberry farms at bed scale. There were 1696 fully documented observations (Table 1) reporting berry yield, cultivar, and tissue nutrient composition.

**Table 1:** Regional provenance and pedigree [6,3,6] of cranberry cultivars in the dataset.

Cultivar	Data collection	Quebec	Wisconsin	Pedigree	Release year	Origin
		# observations				
Native selections						
Ben Lear	Survey	14	12	Native selection	1901	Wisconsin
Howes	Survey	1	0	Native selection	1843	Massachusetts
LeMunyon	Survey	0	13	Native selection	1960	New-Jersey
McFarlin	Survey	0	1	Native selection	1874	Massachusetts
Searles	Survey	0	15	Native selection	1893	Wisconsin
Early cultivars						
Bergman	Survey	10	0	Early Black × Searles	1961	New-Jersey
Pilgrim	Survey	17	10	McFarlin x Prolific	1961	Massachusetts
Stevens	Survey	298	117	McFarlin × Potters Favorite	1950	New-Jersey
Stevens	Fertilizer trials	1042	6	McFarlin × Potters Favorite	1950	New-Jersey
Wilcox	Survey	3	0	Howes × Searles	1950	New-Jersey
Late cultivars						
Crimson Queen	Survey	0	8	Stevens × Ben Lear	2006	New-Jersey
DeMoranville	Survey	2	10	Franklin × Ben Lear	2006	New-Jersey
GH1	Survey	12	51	Rezin × Searles	2004	Wisconsin
HyRed	Survey	0	25	Stevens × Ben Lear	2003	Wisconsin
Recent cultivars						
BG	Survey	0	4	Beckwith × Grygleski Hybrid 1	2012	Wisconsin
Mullica Queen	Survey	0	17	(Howes × Searles) × LeMunyon	2007	New-Jersey
Ruby Star†	Survey	0	5	HyRed × Bergman	2017	Wisconsin
Sundance	Survey	0	3	Stevens × Lear	2011	Wisconsin
Total		1399	297			

^†^https://www.warf.org/documents/technology-summary/P120284US01.pdf.

A single cultivar name was assigned to each observation as the dominant cultivar because 100% genetic purity is rarely attained in commercial stands due to cuttings supplied from production sites rather than pure stands, and to cross-pollinated flowers and their leftover berries [36]. Stands were irrigated to prevent early frost damage and to maintain soil matric potential between −3 and −7 kPa [5].

The 2000–2002 phosphorus fertilization trials were described by Parent and Marchand [37]. Duplicated multi-nutrient on-farm fertilizer trials were conducted from 2014 to 2018 on permanent plots in four sites located in south-central Quebec, Canada. There were five N doses (0, 15, 30, 45, 60 kg N ha^-1^) applied as acidifying ammonium sulfate (21% N) and sulfur-coated urea (24% N, 2% P, 9% K, 5% S) or organic fertilizers (8% N for aminoacids; 6% N, 0.4%P, and 0.8% K for fish emulsions) and four K doses (0, 40, 80, 120 kg K ha^-1^) applied as potassium sulfate (0% K, 14% S) or Sul-Po-Mag (18% K, 9% Mg, 18%S) overlapping the K application range suggested in USA [2]. Where N treatment was 45 kg N ha^-1^ and K treatment was 80 kg K ha^-1^, the P, Mg, Cu and B were applied at rates of 0, 15, or 30 kg P ha^-1^ as triple super-phosphate (20% P) or bone meal (5.7% P), 0 or 12 kg Mg ha^-1^ as Epsom salt (11% Mg), 0 or 2 kg Cu ha^-1^ as copper sulfate and 0 or 1 kg B ha^-1^ as Solubor. The P, Mg, Cu and B treatments were replaced at three sites in 2016 to test N sources and sulfur treatments while applying 15 kg P ha^-1^ as triple super-phosphate (20% P) or bone meal (5.7% P), 12 kg Mg ha^-1^ as Espom salt (11% Mg), 2 kg Cu ha^-1^ as copper sulfate and 1 kg B ha^-1^ as Solubor in 2016 and 2017. Elementary sulfur was applied at rates of 0, 250, 500, and 1000 kg S ha^-1^ on each of the three sites in the spring of 2016 and 2017 to maintain acidic conditions in the soil. Fertilizers were surface-applied manually at four occasions during the season [2,13], as follows: 15% at early flowering (29 June to 2 July), 35% at 50% flowering (July 8 to 11), 35% at 50% fruit set (July 16 to 19) and 15%, 1–2 weeks after the last application. Sites returned to growers’ nutrient management at site #10 in 2017 and 2018, and at the three other sites in 2018. There was thus a large variation in nutrient supply. Berries were harvested by hand in four 30 cm × 30 cm quadrats per plot lined by a squared frame.

### Soil and tissue analyses

Four soil subsamples were collected in the root zone (0–15 cm) then composited in each experimental plot in the spring before applying fertilization treatments. Soils were air-dried and sieved to less than 2 mm to perform soil tests. Grain-size distribution was determined by sedimentation in Bouyoucos cylinder followed by hand-sieving. Bulk density was measured in the 0–10, 10–20, and 20–30 cm layers using the cylinder method. Soi pH was taken in water. Soil series were identified at the site. Minerals (P, K, Ca, Mg, Cu, Zn, Mn, Fe, Al) were extracted using the Mehlich III method [38] and quantified by inductively coupled plasma optical emission spectrometry. Soil C and N were quantified by combustion (Leco-2000 instrument, St-Louis MO). Soil pH was measured in distilled water.

Leaves and stems were collected between August 15 and September 15 across the observational and experimental sites [19,29,30]. Tissues were not cleaned as recommended unless absolutely necessity [39]. One hundred current season’s fruiting and vegetative uprights were sampled randomly per plot and composited, oven-dried at 65°C for 24 h to 36 h, ground to pass through 1-mm sieve, and analyzed for total P, K, Mg, Ca, B, Cu, Zn, Mn, and Fe by plasma emission spectroscopy (ICP-OES) after tissue digestion. Total N was quantified by micro-Kjeldahl digestion or by combustion (Leco-2000 instrument, St-Louis MO).

### Statistical analysis

#### Machine learning

Machine learning models are useful to describe complex living systems phenomenologically from features [32,40]. Features are independent variables such as climatic, edaphic or managerial data, indices or categories, soil tests and tissue tests. The choice of the machine learning model among tens of models depends on the objective of the user. A random forest is a collection of decision trees useful for classification purposes [40]. Adaboost is a forest boosted by sequentially modelling the error of the previous tree, potentially increasing model accuracy.

Model performance can be assessed in different ways such as cross-validation, one-leave-out, and split into training and validation datasets. Cross-validation is data-saving, allowing to process relatively small datasets rapidly. The training set is split into *k* smaller sets, training the model using *k*-1 folds and validating it using the remaining data. A sequence of *k* boolean tests is run by randomly sampling data with replacement. Accuracy is averaged across the *k* outcomes. Hyperparameters are set to maximize model performance. Orange data mining [41] suggests using 5, 10, or 20 folds (*k*). Data selection is stratified to avoid oversampling certain variables and undersampling others.

Machine learning models can predict outcomes by combining a minimum of key features selected to increase R^2^ or decrease RMSE in regression mode relating predicted and actual values, or to increase area under curve (AUC) and classification accuracy (CA) in classification mode about yield cutoff. The model is informative if AUC > 0.7 [42]. In classification mode the confusion matrix returns four quadrants allowing to classify specimens as true negative (high-yielding and nutritionally balanced specimens), false negative (low-yielding but nutritionally balanced specimens), false positive (high-yielding but nutritionally imbalanced specimens), and true positive (low-yielding and nutritionally imbalanced specimens). The CA is computed as number of true negative and true positive specimens shown in the confusion matrix, divided by total number of observations. The number of true negative specimens should be high to allow diagnosing growing conditions at high yield potential under given combinaitons of factors. Where the number of true negative specimens is too small, false negative specimens that are also nutritionally balanced could be considered as additional nutrient benchmarks.

The list of features and target variables documented in the dataset is provided in Table 2. The fertilization features were reported as total seasonal nutrient applications. Climatic data were obtained from the closest Environment Canada meteorological stations within 10 km of the sites. Yield cutoff between high and low yields was set at 40 ton ha^-1^ (above average yield of ≈ 30 ton ha^-1^ in Wisconsin and Quebec) to run the Random Forest model in regression and classification modes using Orange 3.23 [41]. In preliminary analysis, Random Forest performed better than other learners such as Gradient Boosting, Support Vector Machine, Naïve Bayes, KNN and Neural Network in terms of classification accuracy. While yield cutoff of 40 ton ha^-1^ provides high classification accuracy, it could be adjusted to growers’ capacity to reach higher yields or to site conditions leading to lower yield potential. The confusion matrix allows discarding false positive specimens from the calculation of nutrient standards. False positive specimens can bias nutrient norms as in the Diagnosis and Recommendation Integrated System [43] and the boundary line approach [44] due to luxury consumption, contamination or sub-optimum concentrations [45].

**Table 2:** Features and target variables in the Quebec-Wisconsin dataset.

Feature	Description
Region	Quebec, Wisconsin
Farming system	Conventional, organic
Cultivar	See Table 1
Fertilization	N, P, K, Mg, S, Cu, B
Plant tissue	N, P, K, Mg, Ca, B, Cu, Zn, Mn, Fe
Soil test	pH; total C and N; Mehlich3 P, K, Mg, Ca, Cu, Zn, Mn, Fe, Al
Soil grain-size distribution	Clay, silt, very coarse sand, coarse sand, medium sand, fine sand, very fine sand
Soil bulk density	0–10 cm, 10–20 cm, 20–30 cm
Soil series	St-Judes, St-Samuel, Ste-Sophie (Spodosols and Inceptisols)
Temperature	Monthly means (from beginning of May to end of October)
Precipitations	Monthly totals (from beginning of May to end of October)
Target variable	Yield (ton ha^-1^), yield class about yield cutoff of 40 ton ha^-1^

### Latent variables

To account for carryover effects, we used 575 observations on cultivar ‘Stevens’ from the 5-years fertilizer experiments (2014–2018). We removed two sites in 2017 that have been severely damaged by spring frost. Yield-year models were elaborated as follows:

$$Y_{t+1}=f({∁}_{t})$$

$$Y_{t+1}=f({∁}_{t},\;{F_{t},Y}_{t})\;and\;other\;features,$$

Where *t* is current year, *t+1* is next year, *Y* is berry yield, *C* is foliar tissue composition, and *F* is fertilization.

#### Isometric log ratio transformation

The sample space of tissue composition is defined by tissue nutrient concentrations and a filling value (F_v_) computed by difference between measurement unit (1000 g kg^-1^ on dry weight basis) and the sum of nutrient concentrations. Note that total nutrient analysis is an amalgamation of several molecular or ionic forms of the element, some impacting plant metabolic processes more than others [45]. Amalgamation of components is common in compositional data analysis [46].

Parent [34] suggested using isometric log ratios with orthonormal basis to group nutrients into subsets and to compute the Euclidean distance between two compositions. The isometric log ratio (*ilr*) is a log contrast between the geometric means of two nutrient subsets computed as follows [47]:

$${ilr}_{i}=\sqrt{\frac{rs}{r+s}}ln(\frac{G_{r}}{G_{s}})$$

Where *r* and *s* are the numbers of components at numerator and denominator, respectively, and *G*_*r*_ and *G*_*s*_ are geometric means across the *r* and *s* components at numerator and denominator, respectively. Euclidean distance *ε* between defective and successful (*) tissue compositions was computed as follows for a *D*-parts composition [47,48]:

$$\varepsilon =\sqrt{\sum_{i=1}^{D-1}{\left({ilr}_{i}-{ilr}_{i}^{*}\right)}^{2}}=\sqrt{\sum_{i=1}^{D}{\left({clr}_{i}-{clr}_{i}^{*}\right)}^{2}}$$

Centered log ratios (*clr*) made DRIS compositional [49]. In contrast with *clr*, the *ilr* transformation offers the possibility to focus on selected subsets and to compute their Euclidean distance. While the *ilr* variables have orthonormal basis, they are not uncorrelated [50]. Hence, setting apart groups of nutrients does not mean that there is not relationship with other groups of parts.

The successful Euclidean subspaces were called “enchanting islands” in [34] and “ilhas encantadas” or “Humboldtian loci” in [35]. The reference successful specimens provide not only an assessment of “optimal” nutrient concentrations under conditions similar to those of the diagnosed specimen, but also the associated yield and successful fertilization regime at local scale as documented in the dataset.

The perturbation vector can rank nutrients in the order of their limitation to yield as relative shortage or excess. It is a scaling operation between diagnosed (*X*) and reference (*x*) compositional vectors computed as follows [51]:

$$p=X⊖x=\{\frac{N}{N^{*}},\frac{P}{P^{*}},\ldots \}$$

The reference vector is the composition of successful specimens (*) showing close Euclidean distance from that of the diagnosed specimen. The perturbation vector can be interpreted about the ratio of 1, as per example where there is relative excess, $\frac{X}{x^{*}}>1$, or preferably about zero as $\frac{X}{x^{*}}-1>0$,. The perturbation vector differs from the more common weighted distance between the centroid of the compositional hyperellipsoid of performing crops and composition of the diagnosed specimen.

## Results

### Minimum dataset to run the machine learning model

The minimum dataset was searched iteratively by adding or removing yield-impacting features documented in the Quebec-Wisconsin dataset, followed by comparing the accuracy of the ensuing Random Forest models. We added sequentially regions and cultivars, then climatic indices, soil features, tissue tests, and various combinations of features. The most accurate Random Forest model included all documented growth-limiting features (Table 3).

**Table 3:** Factor contribution to ML model accuracy.

Factors	AUC†	CA‡
Region+cultivar	0.563	0.694
Region+cultivar+climate	0.771	0.761
Region+cultivar+soil test	0.699	0.729
Region+cultivar+tissue test	0.844	0.787
Region+cultivar+tissue test+climate	0.861	0.807
Region+cultivar+tissue test+soil test	0.854	0.805
Region+cultivar+tissue test+soil test+texture+density	0.868	0.819
Region+cultivar+tissue test+soil test+texture+density +climate	0.878	0.833
Region+cultivar+tissue test+climate+soil test+stand age	0.885	0.832

^†^Area Under curve;

^‡^Classification accuracy.

The fact that several features must be combined to increase model accuracy makes regional nutrient standards across factors hazardous to apply at local scale. Nevertheless, the quartiles of true negative tissue concentrations across regions and cultivars were generally narrower than published concentration ranges currently used in North America (Table 4).

**Table 4:** Intervals of compatibility of quartile nutrient concentrations in plant tissues (leaves and stems) of true negative specimens across factors compared to published ranges.

Nutrient	First quartile	Third quartile	Davenport et al. [19]
	g kg^-1^		
N	10.0	11.3	9.0–11.0
P	1.0	1.2	1.0–2.0
K	4.9	5.9	4.0–7.5
Mg	1.8	2.2	1.5–2.5
Ca	7.7	9.5	3.0–8.0
B	0.034	0.065	0.015–0.060
Cu	0.003	0.005	0.004–0.010
Zn	0.016	0.025	0.015–0.030
Mn	0.195	0.441	> 0.010
Fe	0.066	0.114	> 0.020

### Biplot analysis and balance design

**Fig 1:**
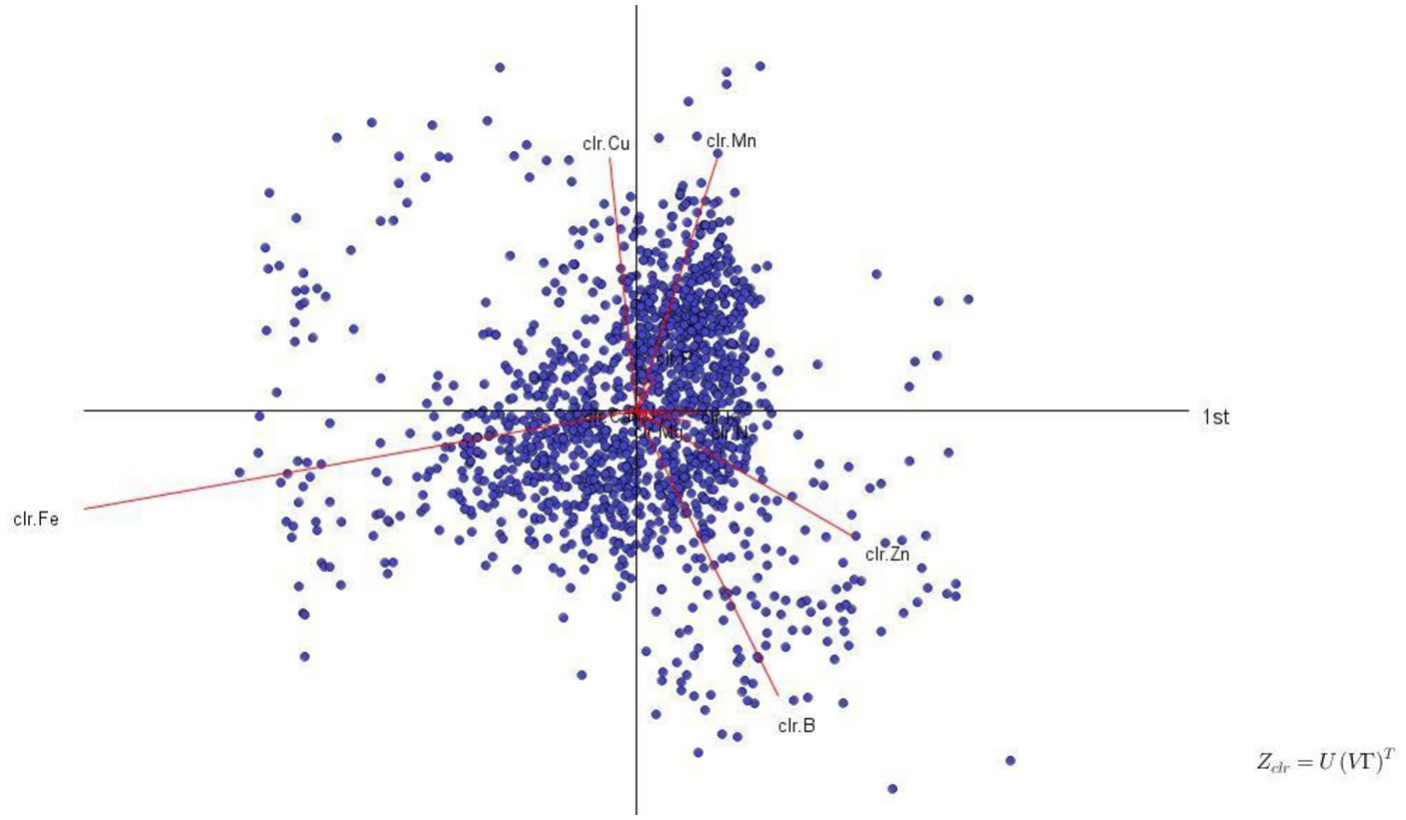
Biplot analysis of the Quebec-Wisconsin dataset.

**Fig 2:**
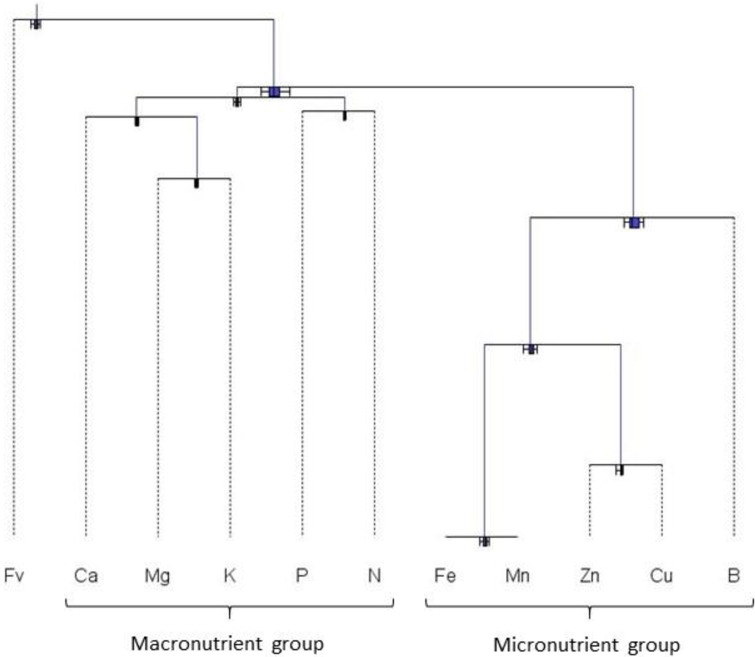
Dendrogram to analyze macro- and micronutrients separately (Fv = filling value).

Biplot analysis of the tissue analytical results showed that micronutrients were much more variable than macronutrients (Fig 1), indicating large variation in local soil mineralogy as well as managerial features such as applications of fungicides and fertilizer micronutrients. The dendrogram in Fig 2 showed that the large variation in micronutrient concentrations impacted considerably the balance between macro- and micronutrients. Due to large variation in concentration values among micronutrients, the perturbation vector should be interpreted with care to avoid diagnosing excessive shortage or excess of micronutrients without additional information on soil test and management practices.

### Local nutrient diagnosis

Yield cutoff between high or low yields was set at 40 ton ha^-1^, but other yield cutoffs may have been selected depending on growers’ objective. The ML classification model returned risk analysis as predicted probability to exceed yield cutoff. Thereafter, the *ilr* variables allowed computing Euclidean distance between the defective and successful compositions. Factor-specific nutrient diagnosis implied comparing, using the perturbation vector, the composition of a diagnosed specimen to that of the nearest successful neighbors among 265 true negative specimens. The perturbation vector ranked nutrients in a numerical order of limitation to yield.

Tissue analyses of two cultivars in Quebec and Wisconsin were diagnosed across the documented features (Table 5). The Random Forest prediction model showed probabilities of 13% and 25% for Quebec and Wisconsin defective specimens to attain high yield (> 40 ton ha^-1^). As a result, both low-yielding specimens were declared nutritionally imbalanced. Tissue compositions of defective specimens were compared to those of the corresponding closest successful specimens to identify the source of nutrient imbalance and the attainable trustful high yields by rebalancing tissue nutrients at local scale. By comparison, the average composition of the 265 true negative specimens at regional scale was also used as regional reference composition.

**Table 5:** Diagnosis of two Quebec and Wisconsin cranberry cultivars against their respective closest Euclidean distances from successful cultivar-specific neighbors.

Region	Cultivar	Yield	N	P	K	Mg	Ca	B	Cu	Zn	Mn	Fe	Fv	Euclidean distance
		ton kg^-1^	g kg^-1^											
		Defective specimens												
Quebec	Stevens	26.7	12.1	1.1	4.0	1.5	8.2	0.030	0.005	0.030	0.220	0.090	972.7	-
Wisconsin	Crimson Queen	11.7	10.2	1.4	6.9	2.0	9.3	0.024	0.004	0.017	1.198	0.060	968.9	-
		Closest successful specimens at local scale												
Quebec	Stevens	57.6	10.1	1.0	4.7	1.9	9.0	0.029	0.006	0.018	0.222	0.097	972.9	0.66
Wisconsin	Crimson Queen	62.8	12.3	1.5	6.2	2.8	14.7	0.053	0.003	0.020	0.339	0.074	962.0	1.64
		Regional averages of true negative specimens												
All regions	All cultivars TN	> 40	10.6	1.1	5.4	2.0	8.9	0.052	0.004	0.021	0.355	0.117	971.3	0.82;1.77†

^†^ Euclidean distance between average composition of TN specimens and that of defective specimens in Quebec and Wisconsin, respectively.

**Fig 3:**
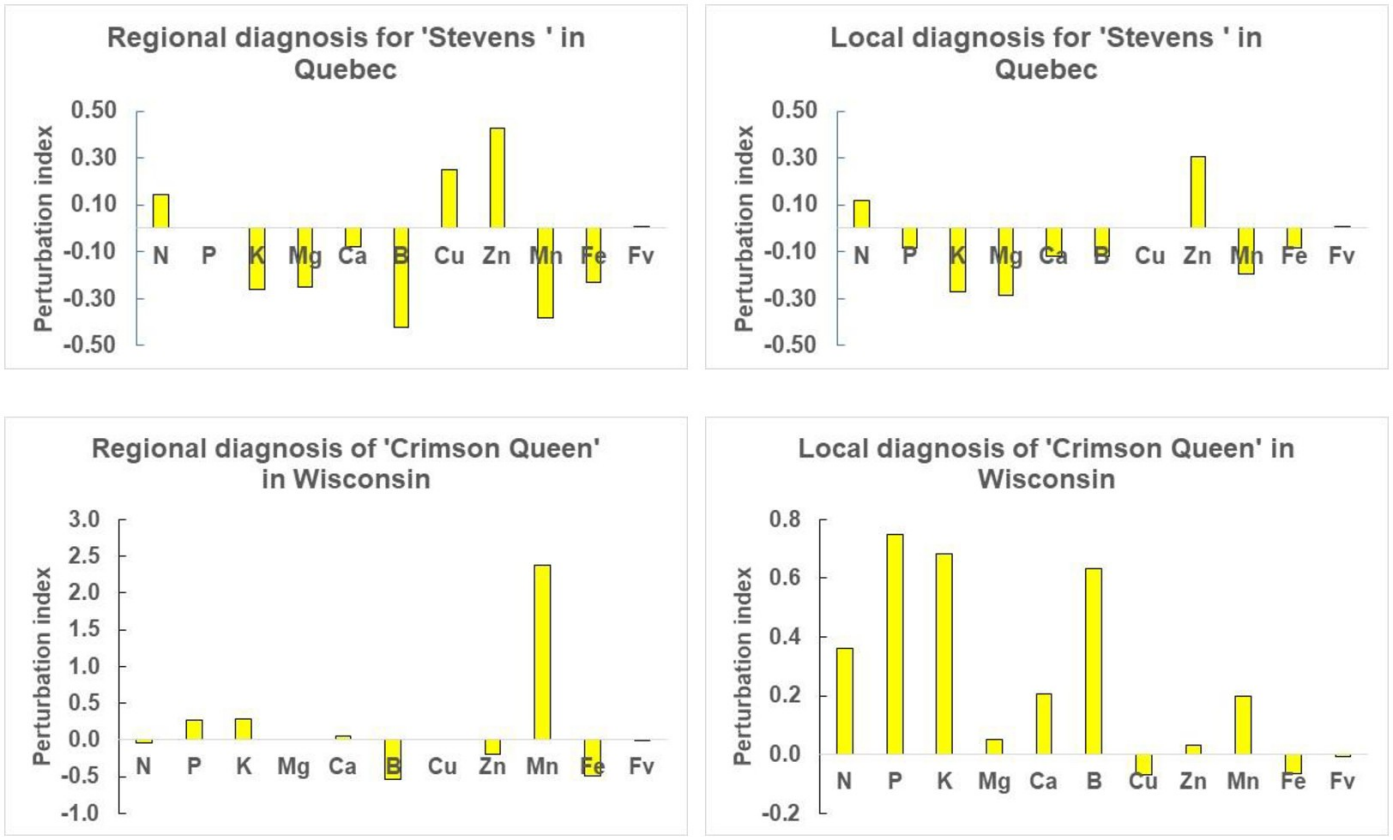
Nutrient diagnosis for ‘Stevens’ in Quebec and ‘Crimson Queen’ in Wisconsin at regional scale across factors or at local scale at factor-specific level.

Regional nutrient diagnosis of cultivar ‘Stevens’ in Quebec indicated possible N, Cu and Zn excess, and K, Mg, B, Mn and Fe shortage to achieve > 40 ton ha^-1^. Local diagnosis detected N and Zn excess, and P, K, Mg, Ca, B, Mn and Fe shortage for attainable yield of 57.6 ton ha^-1^ (Fig 3). Regional nutrient diagnosis of cultivar ‘Crimson Queen’ in Wisconsin indicated B, Zn and Fe shortage and P, K and Mn excess to reach > 40 ton ha^-1^. Local diagnosis returned N, P, K, Mg, Ca, B abd Mn excess and Cu and Fe shortage at local scale where yield potential of the closest neighbor was 62.8 ton ha^-1^ (Fig 3). Euclidean distance between diagnosed and reference compositions were higher using regional average concentrations compared to the closest true negative specimen (Table 5). While regional averages are statistical constructs, compositional entities at local scale are combinations of nutrients uniquely impacted by site-specific genetic х environment х management interactions. Those results showed that adding local factors to diagnose tissue compositions changed the traditional interpretation of plant nutrient status based on regional nutrient references averaged across factors.

### Yield prediction model including latent variables

Current berry yield is a measure of carbohydrate depletion while tissue test is an index of stored nutrients that could be mobilized in the following years. The 2014–2018 experiments provided such information. While berry yields and soil and tissue analyses collected annually on the same beds can grow rapidly in size and diversity with growers’ collaboration, they could be informative to predict future yields if collected in the same plots through time.

**Fig 4:**
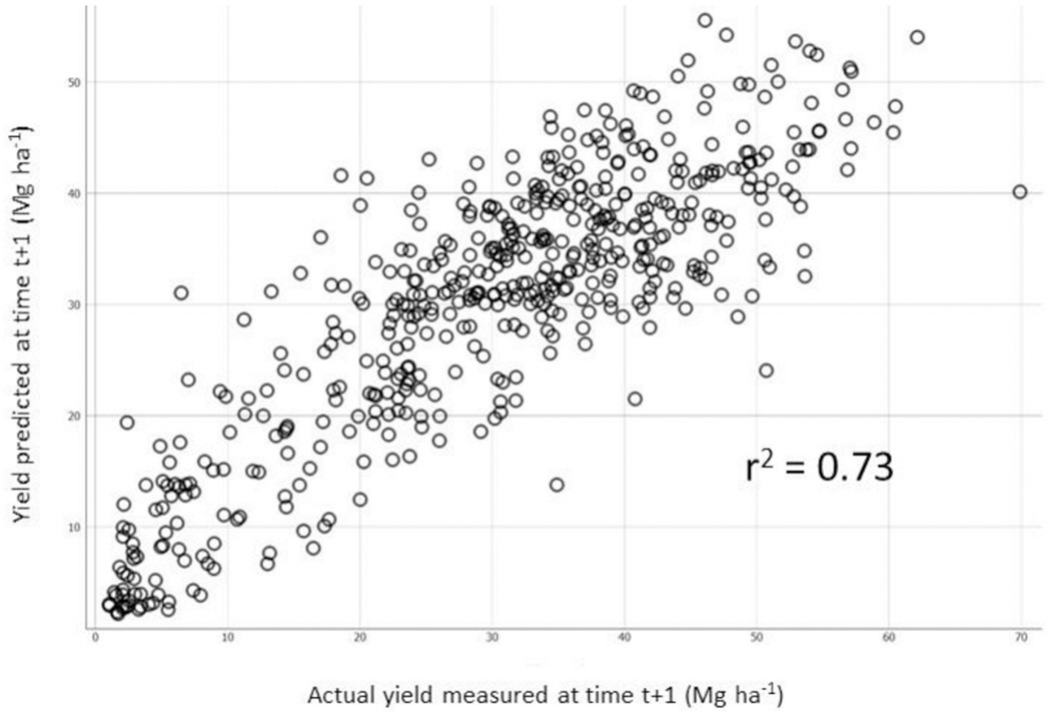
Relationship between actual and predicted cranberry yields at year t+1.

After removing two sites due to severe early frost damage in 2017, 575 observations were retained to predict next-year yields based on current-year yields and features. The R^2^ values of the predictive Random Forest model depended on the number of features included in yield functions (Table 6). Even without future climatic indices at hand, yield prediction for the following year was satisfactory (Fig 4), providing evidence for carryover effects.

**Table 6:** Accuracy of Random Forest regression models (*Y*_*t*_, *Y*_t+1_ for yield, *t* for current year, *F*_*t*_ for fertilization regime, and C_*t*_ for tissue composition) using the Quebec 2014–2018 fertilization trials with cultivar “Stevens”.

Yield function	R^2^
1. Y_t+1_ = f(C_t_)	0.600
2. Y_t+1_ = f(C_t_, F_t_)	0.632
3. Y_t+1_ = f(C_t_, F_t_, Y_t_)	0.639
4. Y_t+1_ = f(F_t_, Y_t_) + soil test	0.684
5. Y_t+1_ = f(C_t_, Y_t_) + soil test	0.687
6. Y_t+1_ = f(C_t_, F_t_, Y_t_) + soil test	0.730

The Random Forest classification model returned similar CA values of 0.848 for current-year and 0.847 for next-year predictions. However, there were 165 true negative specimens for current-year yield assessment, and 64 true negative specimens for next-year yield assessment, indicating mismatch between current-year and next-year true negative specimens.

**Fig 5:**
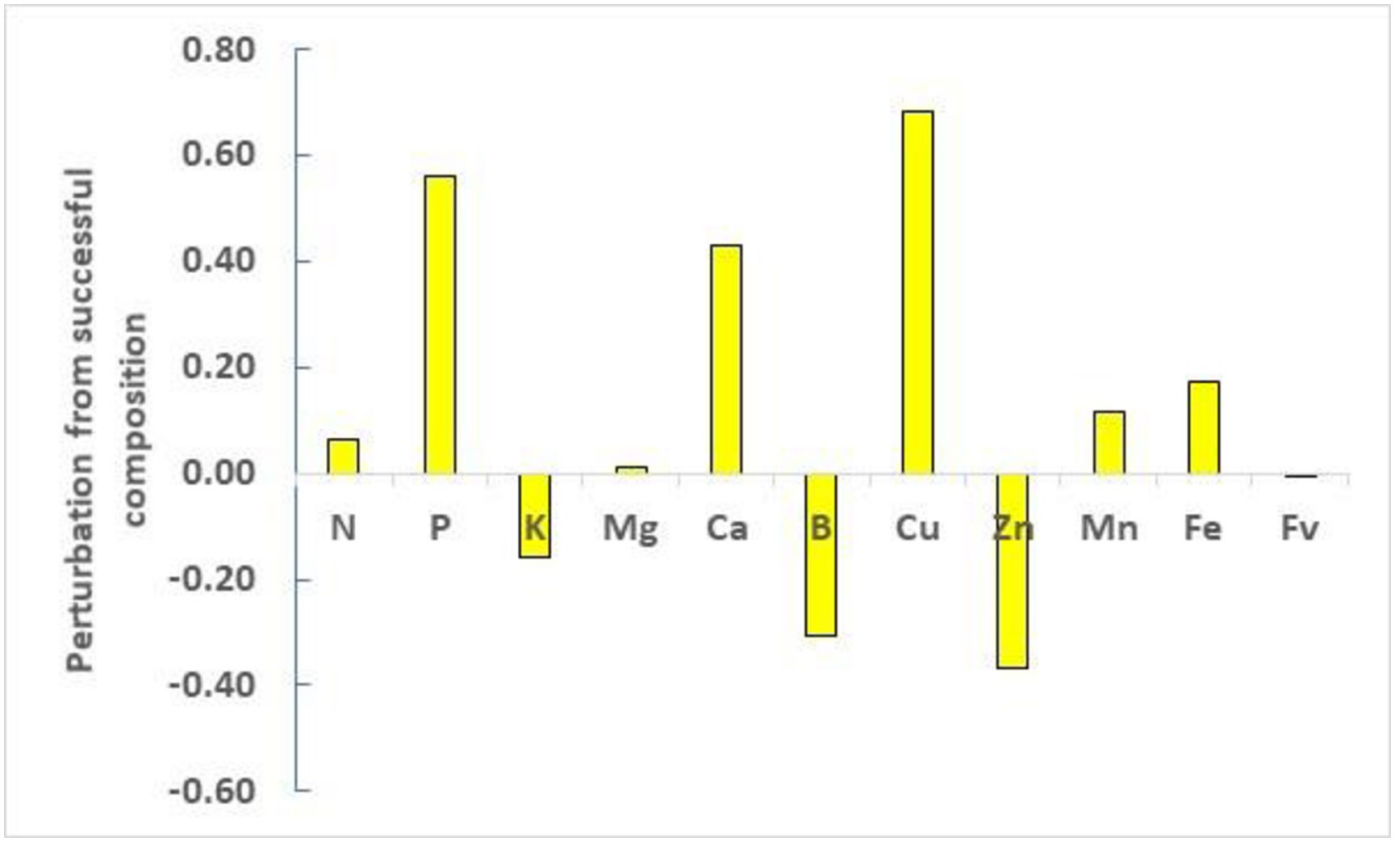
Perturbation vector of the defective Quebec specimen in Table 4 compared to a close successful specimen to attain high yield in the following year.

A predictive diagnosis was conducted for the Quebec defective ‘Stevens’ specimen in Table 6. Among the 64 true negative specimens predicted by the carryover model, a successful specimen predicted to produce 57.1 ton ha^-1^ in the next year showed Euclidean distance of 0.99 and the following concentration values in the current year: 11.4 g N kg^-1^, 0.7 g P kg^-1^, 4.8 g K kg^-1^, 1.5 g Mg kg^-1^, 5.7 g Ca kg^-1^, 0.043 g B kg^-1^, 0.003 g Cu kg^-1^, 0.048 g Zn kg^-1^, 0.197 g Mn kg^-1^, and 0.077 g Fe kg^-1^. The perturbation vector showed relative excess of P, Ca, and Cu, and relative shortage of K, B and Zn (Fig 5). This suggests discontinuing P, Ca and Cu fertilization, and increasing K, B and Zn rates. This emphasizes the importance of collecting large and diversified data on the same plots through time, and to conduct local factor-specific diagnosis across several features against close successful neighbors to increase the probability to attain high yield.

## Discussion

### Nutrient standards

Tissue test interpretation is based traditionally on fixed nutrient sufficiency ranges collected at regional scale and averaged across myriads of genetic х environment х management interactions. Therefore, the diagnostician must assume heroically that all controllable and uncontrollable factors other than those being addressed are similar or at near-optimum levels [52]. Ulrich [53] pointed out that “in view of the great variability of plants and soils, it would be remarkable if all plants became deficient in nutrients at the same time”.

Indeed, growers are used to compare defective specimens to successful neighbors. Parent [34] represented growers searching for successful conditions as compositional parachutists trying to land on the closest “enchanting islands” by manipulating nutrients represented by paracords. Where high yields have been reached in the successful neighborhood, realistic yields can be targeted and trustful correction measures applied. Factor specific diagnosis translates into site-specific recommendations and precision agriculture.

In our study, we assembled experimental and observational cranberry data from Quebec and Wisconsin. We found that relating berry yield to nutrient composition of leaves and stems and other features collected in the same year resulted in the highest model accuracy where all yield-impacting features documented in the dataset were included in the model. There is thus a need for paradigm change toward factor-specific nutrient diagnosis and site-specific fertilizer recommendations supported by large and diversified datasets [35,54,55].

### Carryover effects

While substantial response to fertilization is possible where strong nutrient deficiency occurs, the major contribution of tissue testing is to reduce or discontinue the unneeded application of fertilizers in the following years [14]. Relationships between yield and tissue composition of annual crops have been found appropriate to elaborate nutrient standards [56]. For perennial crops, more modelling effort must be implemented because latent processes to account for carbohydrate depletion and nutrient cycling during preceding years may impact crop productivity thereafter.

Nutrient carryover effects can occur because nutrients can be stored in plant tissues during the year of fertilization [22,23] and rendered available later on for cranberry production [2]. Biennal effects occur where carbohydrate supply limits yield [8]. Based on model accuracy, next-year cranberry yield and composition of leaves and stems proved to be sensitive to tissue test (leaves and stems), applied fertilization and carbon allocation to fruits during the preceding yield in Quebec fertilizer trials with cultivar ‘Stevens’.

Carryover effects on yield prediction and optimum nutrient management have been modelled successfully in the special case of lowbush blueberry (*Vaccinium angustifolium*) because the tissue is sampled the year preceding harvest and the crop is harvested every other year [54]. For most perennial fruit crops, the harvesting is annual. Annual geolocalized sampling is thus recommended to identify nutritional problem in the current year in order to adjust fertilization in the following year for long-term nutrient management [14]. We translated this concept into accurate machine learning and compositional models. Data acquired from on-farm surveys relating soil and tissue analyses to crop performance could thus contribute to document carryover effects from the preceding year for next-year yield prediction and fertilizer recommendation at local scale.

### Acquisition of large data sets

Successful compositional neighborhood sharing the same features but differing in the ones that could limit yield can provide trustful corrective measures tailored for local scale. However, this requires large, informative and diversified datasets to capture numerous combinations of features. While experimental data are expensive to acquire and specific to the experimental areas, on-farm survey data can be collected to build large and diversified datasets as supported by “citizen science projects” [57].

Kyveryga et al. [58] and Anderson and Kyveryga [59] stressed the great importance of historical farm data to search for near-optimum dosage and to guide fertilization decisions. Indeed, more than two hundred years ago, Alexander von Humboldt elaborated the principles of biogeography to document complex interactive biosystems by facts, measurements, and evidence at local scale [33]. Such large datasets can now be solved easily using tools of machine learning and compositional data analysis.

## Conclusion

Well-documented datasets processed by machine learning and compositional methods allow conducting nutrient diagnosis at cultivar × environment × management interaction levels. This is a major change of paradigm compared to traditional diagnostic methods elaborated across growth-impacting factors. The Random Forest model confirmed that carryover effects of carbohydrate and nutrient accumulations impacted berry yield in the following year. This emphasized acquiring large and diversified datasets. Cranberry nutrient datasets could grow rapidly at minimum cost through collaboration between researchers and growers to develop accurate nutrient diagnostic tools. Factor-specific diagnosis must translate into site-specific fertilizer recommendations and precision agriculture.
